# Use of dual-energy computed tomography for the evaluation of calcinosis in patients with systemic sclerosis

**DOI:** 10.1007/s10067-015-2998-7

**Published:** 2015-07-03

**Authors:** Vivien Hsu, Mark Bramwit, Naomi Schlesinger

**Affiliations:** Department of Medicine, Rutgers-Robert Wood Johnson Medical School, New Brunswick, NJ USA; University of Radiology Group, Robert Wood Johnson University Hospital, New Brunswick, NJ USA; Adult Clinical Research Center, 51 French Street, 3rd floor Acute Care Building, New Brunswick, NJ 08903 USA

**Keywords:** Calcinosis, Systemic sclerosis

## Abstract

We examined the usefulness of dual-energy computed tomography (DECT) in the evaluation of symptomatic systemic sclerosis (SSc)-related calcinosis of the hands. We performed DECT scan of the hands in 16 patients with symptomatic SSc-calcinosis to better characterize the calcinosis, their locations within the soft tissues, and exclude monosodium urate (MSU) crystal deposition. We also compared their computed tomography (CT) images to plain radiographs of one hand. Pertinent clinical information from this cohort was collected. Sixteen SSc patients underwent DECT and plain X-ray imaging of the hands. Five of the 16 SSc patients in our cohort had overlap disease, either rheumatoid arthritis (RA) and/or myopathy. Calcinosis symptoms included painful deposits (88 %), soft tissue swelling (66 %), and recurrent infections (44 %) from these deposits. On DECT, calcinosis deposits had the same color and density as the bone and no MSU was found. However, their CT images showed better details of the calcinosis locations in the soft tissues as well as the bone destruction, especially if there was overlying bulky deposits or flexion contractures. These deposits were most commonly found in the subcutaneous fat pads of the fingertips, along tendon and muscle groups, or within the carpal tunnel. DECT did not confirm MSU in our cohort with calcinosis. However, CT imaging was superior to plain radiographs in locating these deposits within the soft tissues and may be a useful tool to study SSc-calcinosis affecting the hands, particularly in the setting of progressive hand deformities.

## Introduction

Calcinosis, the deposition of calcified material in the skin and subcutaneous soft tissues, is frequently seen in patients with symptomatic systemic sclerosis (SSc). It has been reported that approximately 40 % of patients with limited SSc have calcinosis [1, 2]. Calcium hydroxyapatite [3] is reported to be the major constituent of SSc-calcinosis. Its pathogenesis is unknown, and currently, there is no effective cure or prevention. Deposits are commonly found in pressures areas of the elbows, knees, and hands. The hands may be involved in up to 70 % of those with SSc-calcinosis [3]. SSc-calcinosis can be asymptomatic or cause significant morbidity such as pain, intractable ulcers, and recurrent infections. SSc-calcinosis may be palpable on exam and/or diagnosed by imaging. Plain X-rays [4] are useful to confirm location of calcinosis and could be used to estimate the area of some of these deposits, whereas ultrasound [2, 3] and computed tomography (CT) [3, 5] are more helpful in showing the location of these deposits in the soft tissues and could be useful to quantitate the burden of calcinosis using volumetric measurements of many of these bulky and irregularly shaped deposits. Using standard CT with specialized software to permit dual-energy CT techniques, dual-energy computed tomography (DECT) is an advanced imaging modality useful for assessing monosodium urate (MSU) crystal deposition in gout [6–8]. The DECT utilizes two energy beams, usually a combination of 80- and 140-kilovoltage peak (kVp) beams, and differences in attenuation enable differentiation between calcium hydroxyapatite and MSU crystals. Thus, DECT enables visualization of calcium deposits by analysis of the chemical content of scanned materials.

## Methods

In this IRB-approved study, we performed DECT imaging with a 64-slice dual-source CT (Siemens Definition), using dual-energy application in patients with symptomatic SSc-calcinosis, to better characterize the location and radiographic details of SSc-calcinosis and exclude MSU. We also compared the DECT to plain X-ray radiographs in all 16 patients. All had known calcinosis confirmed by prior imaging or physical exam and met the American College of Rheumatology (ACR) criteria for definite SSc [9–11], either limited or diffuse cutaneous SSc. Hand symptoms included painful swelling surrounding the deposits, redness, or recurrent ulceration from draining calcinosis. Variables were precisely defined; standardized abstraction forms were used for data collection. The variables included gender, age, type of SSc, presence of overlap with other rheumatic diseases [12, 13] mean years of SSc, mean years of calcinosis, ischemic digital ulcer history, nailfold capillaroscopy (using portable DermLite DL100), calcinosis complications, calcinosis locations, and autoantibodies.

## Results

The clinical, demographic, and serologic characteristics of our SSc patient cohort are summarized in Table 1. Sixteen SSc patients with symptomatic calcinosis of the hands confirmed by physical examination and/or prior imaging underwent DECT and plain X-ray imaging of the hands. Common calcinosis symptoms included pain at the site of deposits (88 %), soft tissue swelling (63 %) or recurrent infections (44 %) near the deposits. Their mean age was 59.3 (SD 14.6229, range 29–85) and mean disease duration (from onset of non-Raynaud symptoms) was 18.5 years (SD 10.0995, range 7–43 years). Nine of the 16 patients (56 %) had diffuse SSc. Five SSc patients had overlap disease: three with rheumatoid arthritis (RA), one had both RA and myopathy, and the other had SSc with myopathy. Most (12/16 or 75 %) developed calcinosis later in their disease course (mean duration of calcinosis 12.333 years (SD 10.0971, range 1–30)). A long history of ischemic digital ulcers (69 %) was common.

**Table 1:** Clinical and DECT characteristics in SSc-calcinosis cohort

Clinical characteristics	Number of patients (%) (*n* = 16)
Race	
Caucasian	13 (81 %)
African-American	3 (19 %)
SSc type	
Diffuse	9 (56 %)
Limited	7 (44 %)
SSc patients with overlap disease	5 (31 %)
SSc with RA	3
SSc with RA and myopathy	1
SSc with myopathy	1
Mean age	59.3 (SD ± 14.6229)
Mean years of SSc from non-Raynaud	18.5 (SD ± 10.0995)
Mean years of calcinosis	12.33 (SD ± 10.0971)
Ischemic digital ulcer history	11 (69 %)
Digital pulp loss	12 (75 %)
Abnormal nailfold capillaroscopy	8 (50 %)
Calcinosis complications	
Pain	14 (88 %)
Draining ulcer(s)	11 (69 %)
Episodic swelling near deposits	10 (63 %)
Infection	7 (44 %)
Serology	
Antinuclear antibodies (ANA)	14 (87 %)
Anti-topoisomerase (Scl 70)	7 (44 %)
Anti-centromere (ACA)	4 (25 %)
Nucleolar antibody	4 (25 %)
Anti-ribonucleoprotein (RNP)	3 (19 %)
RNA polymerase 3	2 (13 %)
Details seen on DECT scan	
Calcinosis locations	
Fingertip padding	15 (94 %)
Hands	13 (81 %)
Wrists	8 (50 %) (5 in carpal tunnel)
Forearms	5 (31 %)
Presence of acro-osteolysis	15 (94 %)
Calcinosis near acro-osteolysis site	9 (56 %)

**Fig. 1:**
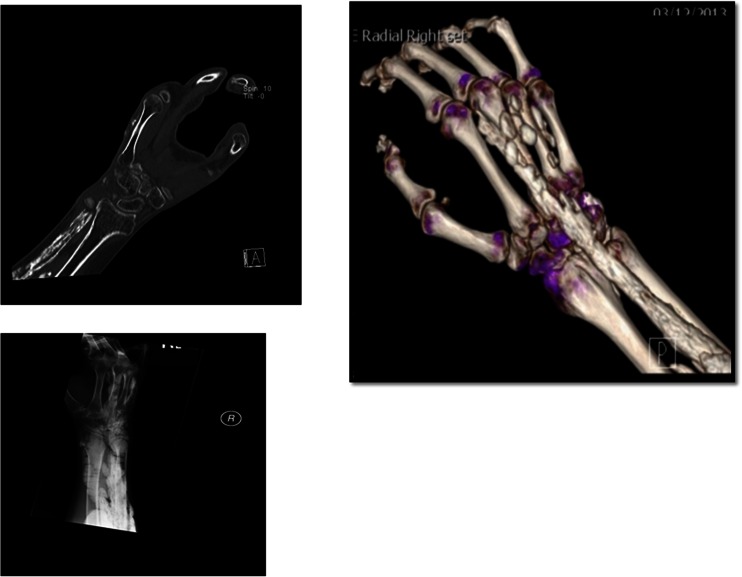
There is extensive soft tissue calcinosis in the visualized portion of the distal forearm as well as within the dorsum of the right hand. There is also acro-osteolysis of the fingers with calcinosis in the soft tissues of the fingertips. On the CAT scan, dorsal calcinosis in the forearm appears to be intramuscular; there are also extensive calcifications in the subcutaneous fat along the volar aspect of the forearm and within the dorsum of the right hand

**Fig. 2:**
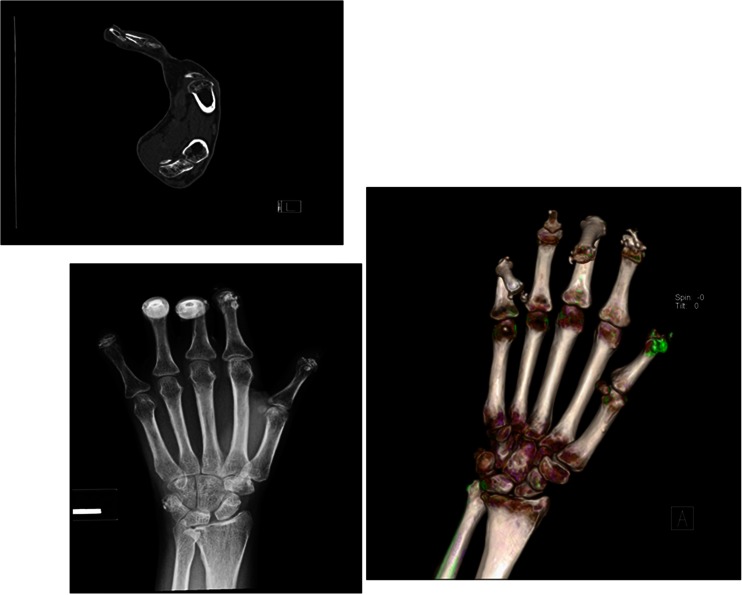
AP view of right hand reveals flexion deformities, acro-osteolysis, and calcinosis of all five fingers. A representative sagittal CT image with bone windows and 3-D volume rendered CT of the third finger shows a clearer example of the findings

**Fig. 3:**
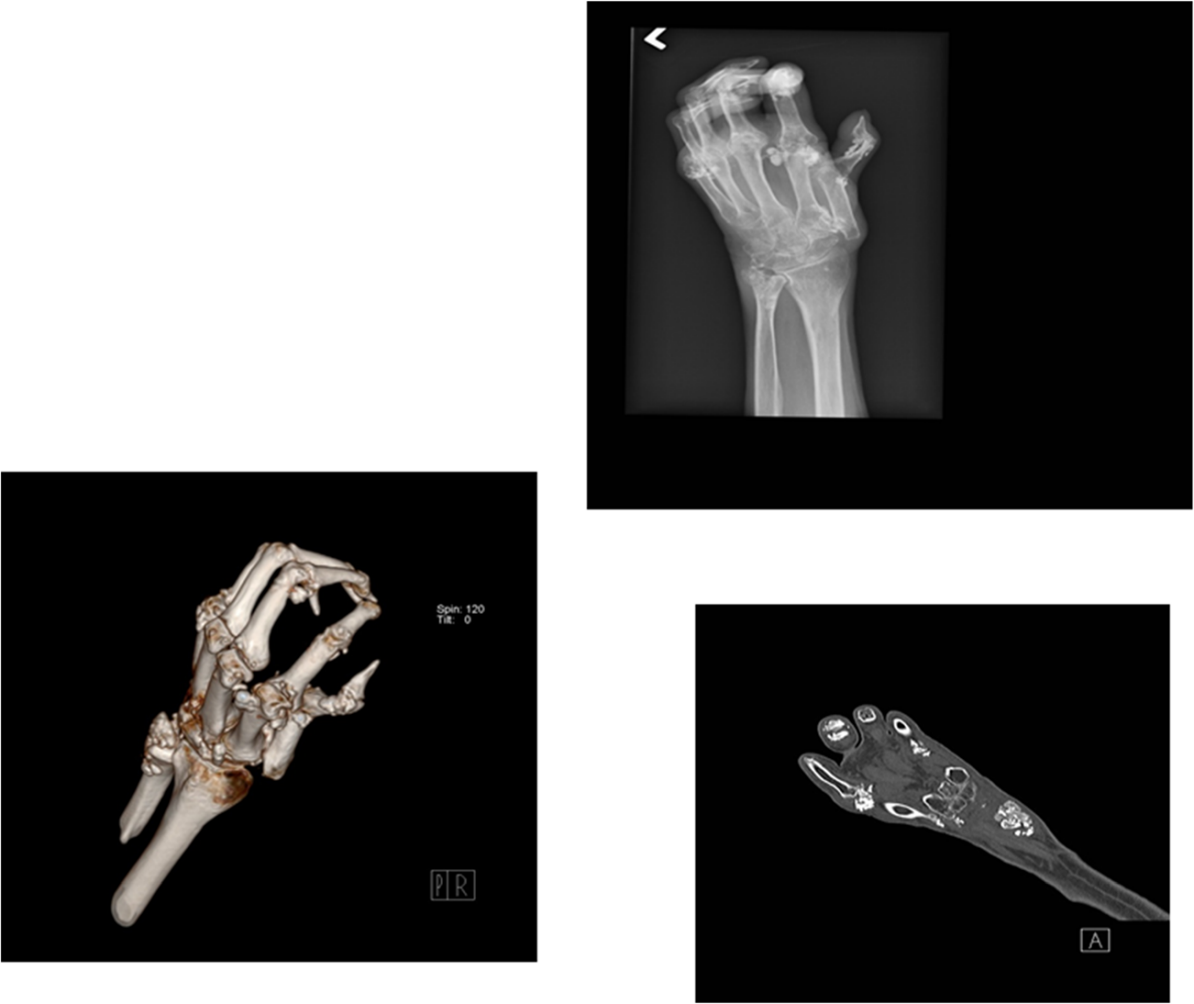
Oblique volume rendered 3-D CT, a single image from the coronal CT with bone windows, and an oblique radiograph of the left hand are shown: CT imaging shows better views of the extensive calcinosis surrounding the carpal bones, distal ulna, metacarpophalangeal (MCP) joints, and interphalangeal joints and extensive periarticular erosions with destructive changes of the articular cortical surfaces. CT sections show some calcinosis deposits deep to the tendons, others are attached to tendons. There are flexion deformities of all fingers and diffuse subluxations involving the MCP joints and first carpometacarpal joint

DECT confirmed calcinosis had the same density and color as the bone, and no MSU was found within or near any of these tumorous deposits. Common locations (see Table 1) included the subcutaneous fat pad of the fingertips, along tendon sheaths of the hands and wrists, within the carpal tunnel, and adjacent to muscle groups. These deposits ranged in size and shapes, some rounded, lobulated (popcorn-like), or in clusters and sheets following tendon and muscle groups (see Figs. 1, 2, and 3). Acro-osteolysis was quite common (94 %), and more than half of this cohort (9/16 patients) had clumps of calcinosis adjacent to their sites of bone destruction.

Plain X-ray imaging of the hands was sufficient to confirm and assess the size and distribution of calcinosis in most of our cohort, although in five subjects the DECT showed better details due to significant flexion deformities or osteopenia in the presence of large and extensive calcinosis nearby. Figures 1, 2, and 3 compare representative DECT images with their plain radiographs of three subjects with significant hand deformities. Figures 1 and 2 were taken from two subjects with diffuse cutaneous scleroderma, both presented with increasingly painful flexion contractures of the hands. Their extensive calcinosis was confirmed incidentally by imaging. Figure 3 was a subject with limited cutaneous scleroderma and erosive arthritis, whose extensive joint destruction and bulky calcinosis were better demonstrated on DECT.

## Discussion

We chose DECT to study SSc-calcinosis because of its capacity to show color produced by MSU deposition. If present, we expected MSU could be detected in the soft tissues, along tendons or ligaments [7, 8], or perhaps as a component within the calcinosis. All but three of our subjects were older and had risk factors for gout, although never symptomatic. However, no MSU deposition was seen and the calcinosis appeared as white and dense as the bone on DECT.

Calcinosis has been reported to be more common in limited cutaneous SSc [1, 2], although most of our cohort with symptomatic SSc-calcinosis had diffuse SSc. Patients with more than 10 years of SSc-calcinosis typically had limited cutaneous SSc. These deposits were typically found along extensor surfaces of the hands, elbows, and knees, although rare locations such as the spine and anterior neck [3, 14] have been reported. Depending on location and size, calcinosis can affect quality of life and may be inflammatory in up to 20 % of patients [3, 15].

Calcinosis in SSc-overlap with myopathy can be tumorous and found in similar locations in the soft tissues [3]. Both SSc patients with myopathy from our cohort had advanced SSc of more than 10 years duration. One patient had no knowledge of her calcinosis until X-rays were done to assess her worsening hand deformities (see Fig. 2). We found both SSc-myopathy patients had similar appearing calcinosis (limited to their hands) as the others without overlap disease.

Acro-osteolysis of the fingertips was common. Johnstone and others [15] reported more severe calcinosis and acro-osteolysis in patients with tissue damage due to severe digital ischemia and suggested that this may be a marker of more severe disease. Similarly, nailfold capillaroscopy has been helpful in predicting more severe scleroderma complications including digital ulcers from vasculopathy [16]. We found abnormal nailfold capillaroscopy in only half of our cohort.

Crystal composition of calcinosis in SSc patients has previously been identified as calcium hydroxyapatite [3], and the pathophysiology of how or why these are formed in SSc is unknown. Increased production of tumor necrosis factor α, interleukin (IL)-1, IL-6, and other pro-inflammatory cytokines has been reported in patients with SSc and systemic lupus erythematosus who have calcinosis [17, 18].

Calcinosis in our patients had a predilection for SQ fat, attached or near tendon sheaths and along muscle plains. Most (88 %) patients reported pain (to palpation or with activity) at the site of their deposits, even if the area was not draining, which often affected their activities of daily living. Ten patients reported soft tissue swelling near the calcinosis site(s) just prior to spontaneous draining. We found that plain X-ray imaging of the hands was sufficient to view the size and distribution of calcinosis in most of our cohort, although in five subjects CT imaging showed better details due to extensive flexion deformities or osteopenia with nearby large calcinosis deposits. CT imaging found more destructive bone and joint disease in our four SSc patients with rheumatoid arthritis and calcinosis. Their calcinosis was otherwise similar in distribution, size, and extent as the rest of our cohort. Interesting, although their arthritis appeared well controlled clinically, all 4 SSc patients reported progressive and disabling hand deformities over the years. We found CT showed better 3-D details of calcinosis locations within the soft tissue structures and demonstrated more clearly how these could contribute to joint contractures or muscle atrophy. Thus, standard CT imaging should be considered in the evaluation of any SSc patient with progressive hand deformities, especially in the presence of bulky calcinosis, while DECT could be applied if MSU was suspected. Newer software is currently being developed to better differentiate between calcinosis and bone on standard CT, which would be very useful to quantify bulky calcinosis using volumetric measurements.

To our knowledge, we are the first group to use DECT to analyze calcinosis in a cohort of scleroderma patients. Our limitations include the small population of patients, five of whom also had overlap disease. However, their calcinosis did not appear any different or any more extensive than in those without overlap disease, and further studies are needed to determine the differences in pathophysiology between those with and without overlap disease.

## Conclusion

Although no MSU crystals were confirmed by DECT in our cohort, CT imaging is a more useful tool to study patients with bulky SSc-calcinosis affecting the hands.
